# Recanalization of the occluded radial artery via distal transradial access in the anatomic snuffbox

**DOI:** 10.1186/s12872-021-01890-1

**Published:** 2021-02-02

**Authors:** Feng Li, Gan-Wei Shi, Bi-Feng Zhang, Xiao-Long Yu, Hao-Min Huang, Jian-Qiang Xiao, Gao-Jun Cai

**Affiliations:** 1 Department of Cardiology, Wujin Hospital Affiliated To Jiangsu University, The Wujin Clinical College of Xuzhou Medical University, Changzhou, 213017 Jiangsu Province China; 2 Department of Pathology and Molecular Medicine, McMaster University, Hamilton, ON L8S4L8 Canada; 3 Department of Ultrasonic, Wujin Hospital Affiliated To Jiangsu University, The Wujin Clinical College of Xuzhou Medical University, Changzhou, 213017 Jiangsu Province China

**Keywords:** Transradial access, Distal transradial access, Anatomic snuffbox, Radial artery occlusion, Case report

## Abstract

**Background:**

Radial artery occlusion is a common complication after coronary angiography and percutaneous coronary intervention via the transradial access. In recent years, coronary angiography and percutaneous coronary intervention via the distal transradial access has gradually emerged, but recanalization of the occluded radial artery through the distal transradial access has rarely been reported.

**Case presentation:**

A 67-year-old female with arterial hypertension and diabetes mellitus was admitted to the hospital due to chest pain for three hours. She was diagnosed with acute myocardial infarction. After admission, the patient successfully underwent emergency coronary angiography and percutaneous coronary intervention through the right transradial access. Radial artery occlusion was found after the operation, and recanalization was successfully performed through the right distal transradial access before discharge. Immediately after the operation and one month later, vascular ultrasonography showed that the antegrade flow was normal.

**Conclusions:**

This report presents a case of radial artery occlusion after emergency coronary angiography and percutaneous coronary intervention in which recanalization was successfully performed through the right distal transradial access. This case demonstrates that recanalization of a radial artery occlusion via the distal transradial access is safe and feasible.

## Background

The transradial access (TRA) is currently the routine access for coronary angiography (CAG) and percutaneous coronary intervention (PCI), and radial artery occlusion (RAO) is a common complication [1]. In recent years, the distal transradial access (dTRA) (anatomic snuffbox (AS)) has emerged a new access for CAG and PCI [2], and recognition of its advantage in clinical applications by cardiac interventionists has gradually increased. An increasing number of cardiac intervention centers are trying to use this access for CAG and PCI, but there are fewer reports on its use in recanalization of RAO after CAG and PCI. Recently, we encountered a patient with RAO after PCI, and recanalization of the occluded radial artery was successfully performed through dTRA. The patient provided informed consent for publication of this case. The present study was approved by the Ethics Committee of Wujin Hospital affiliated with Jiangsu University (Ethics Committee number: 2019-31).

## Case presentation

**Fig. 1:**
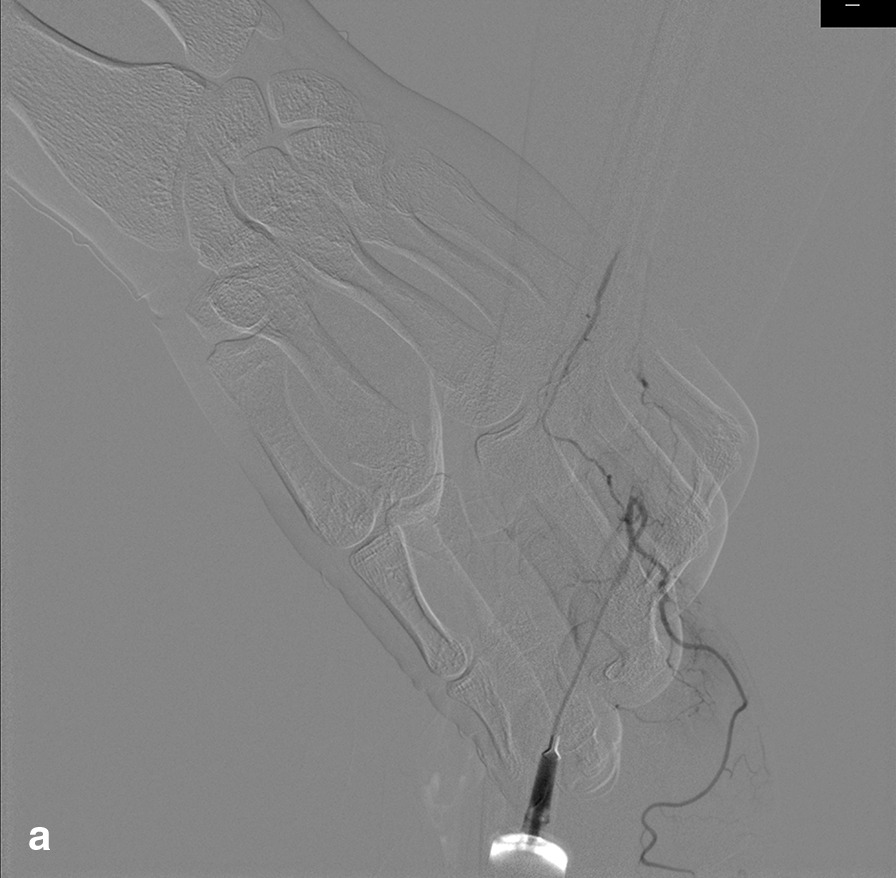
Angiogram via cannula

**Fig. 2:**
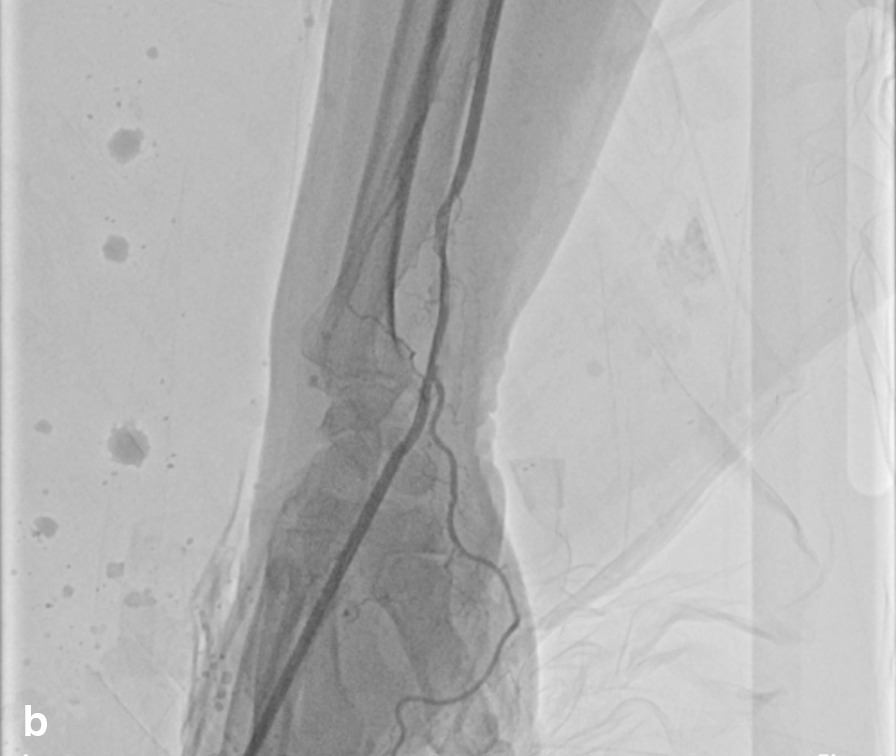
Angiogram of the radial artery resumed

**Fig. 3:**
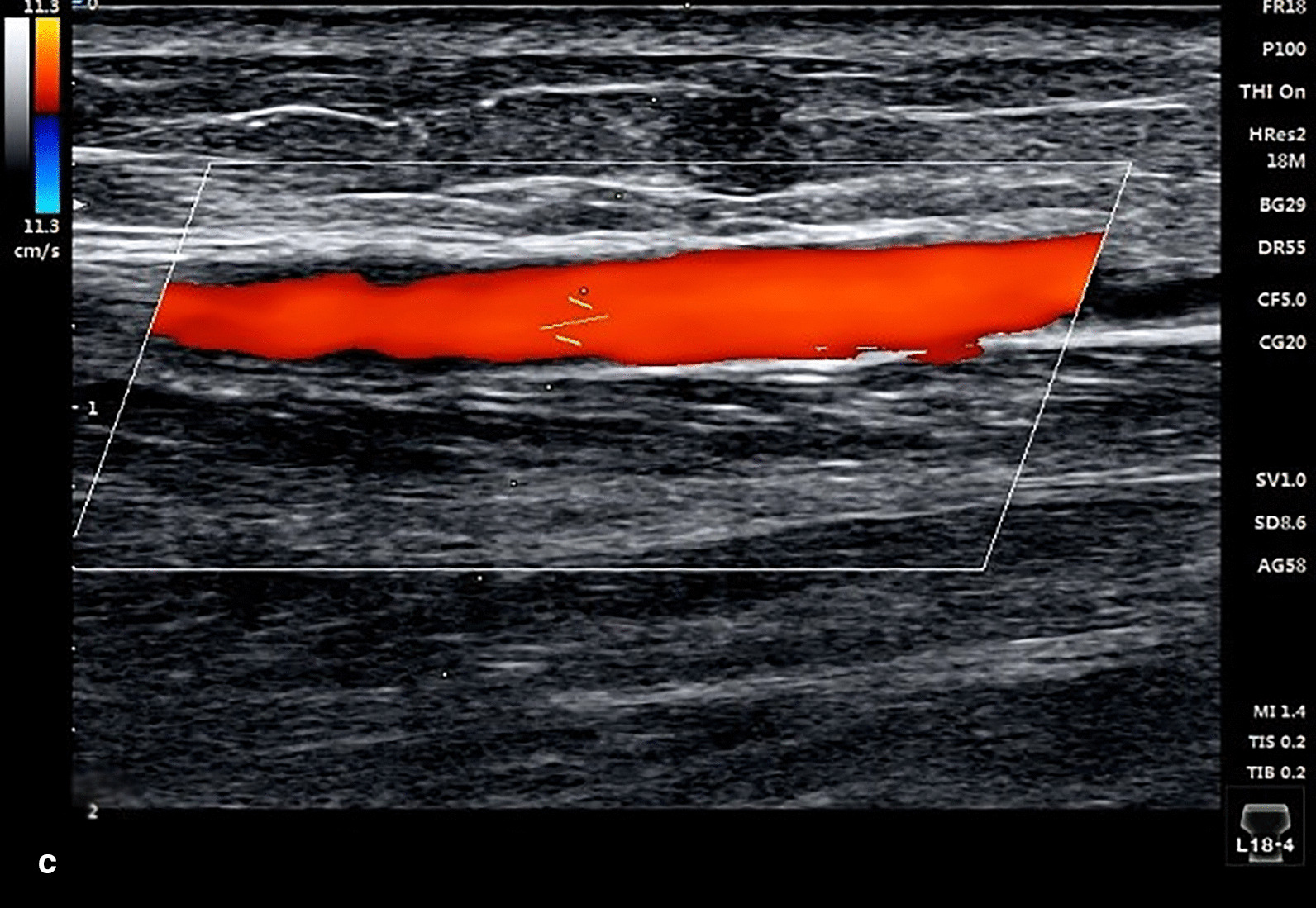
Ultrasonography of the blood flow resumed in the site of radial artery

A 67-year-old female was admitted to the hospital due to chest pain for three hours. She had a history of arterial hypertension for 30 years and diabetes mellitus (DM) for 3 years. ECG showed acute inferior and right ventricular myocardial infarction. After administration of 300 mg aspirin, 180 mg ticagrelor and 5000 U heparin, CAG was carried out emergently via the right TRA. The results showed that there was 60–70% stenosis in the middle of the right coronary artery, and the distal segment was completely occluded. Then, a 2.5 × 28 mm drug-eluting stent was implanted into the distal segment of the right coronary artery. The 6Fr sheath (Terumo Radifocus) was removed from the radial artery after 25.5 h, and the puncture site was bandaged with gauze for ten hours. On the third day post operation, we found that the pulsation of the artery near the puncture site could not be appreciated during palpation, whereas a weak pulse existed in the distal radial artery in the AS region. We suspected that the radial artery might be occluded. Vascular ultrasonography showed that there was no blood flow near the puncture site. Because the patient had recently undergone right RAO, we decided to recanalize the occlusion via the right dTRA. Therefore, after successful puncture of the distal radial artery in the AS, the radial artery was confirmed to be completely occluded by cannula angiography (Fig. 1). Then, a 6Fr sheath (Terumo Radifocus) was inserted into the distal radial artery, and the tip of the sheath was near the distal segment of the occlusion. After failing to absorb the thrombosis, a *Runthrough* guidewire was sent through the occluded segment, then we injected 2000 U heparin and 200 ug nitroglycerin through the sheath, and a BRAUN 2.0 × 20 mm balloon was used to dilate the occluded segment with 8–10 atm × 1 min of pressure for several rounds. Repeated angiography showed that the blood flow of the radial artery was resumed (Fig. 2). The distal radial artery puncture site of in the AS was compressed with gauze for four hours. There were no local complications in the AS region after the operation, such as bleeding, swelling and numbness. The pulsation of the right distal radial artery and the radial artery was normal, and vascular ultrasonography showed that the antegrade flow was normal (Fig. 3). The results of vascular ultrasonography follow-up one month after the operation were the same as those immediately after operation.

## Discussion and conclusions

Currently, the TRA has been recommended as a routine pathway for CAG and PCI [1]. Compared with the femoral artery access, PCI via TRA can reduce the incidence of major cardiovascular adverse events and all-cause mortality in patients with acute coronary syndrome [3, 4]. In recent years, RAO has become a common complication in the TRA, which has received increasing attention from interventional cardiologists. Research have shown that the incidence of RAO is 1%-12% [5, 6] and can be as high as 30% [7]. Many factors might be associated with RAO, such as female sex, DM, sheath size, operation time, low body mass index, long compression time, and repeated operation [8]. Due to the dual blood supply, most patients suffering from RAO have no symptoms. However, RAO limits the utilization, such as for repeated CAG and PCI, of the radial artery, which is used as the bypass grafting and arteriovenous fistula vessel. Therefore, it has recently become increasingly important to repair RAO. For this case, it was a female with DM and long compression time, which might have contributed to the RAO. In addition, in our center, patients with AMI undergoing emergency PCI routinely retain the sheath for monitoring the intra-arterial pressure after procedure. It is also one of the causes of RAO, but it is our local practice and not widely adopted. We have recognized this problem. Recently, we routinely carried out coronary angiography and intervention via the dTRA in our center. The sheath was removed immediately after the procedure, and the hemostatic time was reduced to 3–4 h, which was significantly shorter than before.

In 2017, the dTRA was first described by Kiemeneij as the alternative access for cardiac catheterization [2]. Since then, the safety and efficacy of CAG and PCI via the dTRA has attracted the attention of cardiologic interventionalists [9–11]. At the same time, some studies have found that it was also safe and feasible to recanalize radial artery stenosis or occlusion via dTRA [12–14]. In 2018, Schulte-Hermes et al. [12] successfully performed angioplasty via dTRA in eight cases with severe radial artery stenosis. Sheikh AR et al. reported their successful experience in recanalizing the left RAO through the left dTRA [13]. Under the guidance of vascular ultrasonography, Alkhawam H et al. successfully recanalized chronic RAO in a patient with acute myocardial infarction (AMI) [14]. We first reported the recanalization of RAO via dTRA in a Chinese patient with AMI. In this case, because the RAO occurred recently, the thrombosis could not be absorbed through the sheath. Therefore, we could easily pass through the occluded segment by using a 0.014′’ guild wire during the operation and successfully complete the angioplasty. If the radial artery was occluded for a prolonged time, a strong guide wire might be used [13]. Although the radial artery was occluded, pulsation of the distal radial artery could be appreciated in the AS due to the palmar arches and abundant lateral branches [15]. Furthermore, we are experienced in distal radial artery puncture, which provided a guarantee for successfully repairing the RAO. As we know, the distal radial artery was smaller, which increased the difficulty of the puncture, and successful puncture was the first and the key step to ensure completion of the recanalization. To improve the success rate of distal radial artery puncture, we suggest that puncture should be carried out by experienced cardiologists under the guidance of vascular ultrasonography.

## Data Availability

All available information is contained within the present manuscript.

## Abbreviations

DM: Diabetes mellitus
AMI: Acute myocardial infarction
ECG: Electrocardiogram
CAG: Coronary angiography
TRA: Transradial access
dTRA: Distal transradial access
AS: Anatomic snuffbox
RAO: Radial artery occlusion
PCI: Percutaneous coronary intervention
